# 10-Year Clinical and MRI-Based Outcomes of a Randomized Controlled Trial Evaluating a 6-Week Return to Full Weightbearing After Matrix-Induced Autologous Chondrocyte Implantation

**DOI:** 10.1177/23259671251383136

**Published:** 2025-10-21

**Authors:** Jay R. Ebert, Peter K. Edwards, Sven Klinken, Michael Fallon, David J. Wood, Gregory C. Janes

**Affiliations:** † School of Human Sciences (Exercise and Sport Science), University of Western Australia, Perth, Western Australia; ‡ HFRC Rehabilitation Clinic, Perth, Western Australia; § School of Allied Health, Curtin University, Perth, Western Australia; ‖ Perth Radiological Clinic, Perth, Western Australia; ¶ School of Surgery (Orthopaedics), University of Western Australia, Perth, Western Australia; # Perth Orthopaedic and Sports Medicine Centre, Perth, Western Australia; Investigation performed at the University of Western Australia, Perth, Western Australia

**Keywords:** matrix-induced autologous chondrocyte implantation, partial weightbearing, rehabilitation, clinical outcomes, magnetic resonance imaging

## Abstract

**Background:**

Matrix-induced autologous chondrocyte implantation (MACI) has demonstrated encouraging outcomes in treating symptomatic knee cartilage lesions. Rehabilitation is imperative to optimize outcome, although it has been traditionally conservative.

**Purpose:**

To investigate the longer term clinical and radiological outcome of MACI and investigate any differences in outcome between patients randomized to a 6-week (vs 8-week) return to full weightbearing (WB) after MACI.

**Study Design:**

Randomized controlled trial; Level of evidence, 1.

**Methods:**

A total of 35 patients were recruited into the study, though 37 knees (i.e. two patients had both knees inlcuded at different timepoints) were independently evaluated preoperatively and at 1, 2, 5, and 10 years after surgery. A comparison was made between recruited knees prospectively randomized to a 6-week (n = 18) or 8-week (n = 19) return to full WB after MACI. Patient-reported outcome measures (PROMs) were assessed utilizing the Knee injury and Osteoarthritis Outcome Score. Peak isokinetic knee extensor and flexor torque and single-leg hop capacity (single horizontal, lateral, and medial hop tests for distance) were assessed, with limb symmetry indices (LSIs) calculated. High-resolution magnetic resonance imaging (MRI) was undertaken at all postoperative time points to assess pertinent parameters of graft integrity as per the MOCART (magnetic resonance observation of cartilage repair tissue) system. A combined MRI composite score was also evaluated.

**Results:**

At 10 years after surgery, 31 knees (84%) were available for review, with 3 knees (6 weeks, n = 2; 8 weeks, n = 1) lost to follow-up and 3 knees (6 weeks, n = 1; 8 weeks, n = 2) having already had total knee arthroplasty (TKA) performed before 10 years. All PROMs (apart from the Mental Component Summary of the 36-item Short Form Health Survey [*P* = .57]) significantly improved (*P* < .0001) over the 10-year period, with no group differences (*P* > .05) observed. At 10 years, overall satisfaction was reported as 93% (6 weeks) and 88% (8 weeks). The peak knee extensor torque LSI significantly improved (*P* < .0001) over time, with mean LSIs of 100.8% (6 weeks) and 99.1% (8 weeks) at 10 years. No group differences (*P* > .05) were observed in hop test LSIs, with 10-year hop test LSIs ranging from 99.1% to 103.8%. No significant changes (*P* > .05) were observed for any graft parameter, or the MRI composite score, from 1-year to final 10-year review. Apart from a significant group effect (*P* = .03) for graft tissue intensity in favor of the 6-week group suggesting repair tissue more reflective of native cartilage, no other MRI-based differences (*P* > .05) were reported. At 10 years, 1 further graft (8 weeks) on MRI had failed and, combined with the 3 TKAs, an overall 10-year failure rate of 11.8% was observed.

**Conclusion:**

MACI provided sustained clinical and MRI-based outcomes in most patients to 10 years after surgery, with high satisfaction levels. The 6-week WB protocol did not jeopardize the early or longer term graft outcome.

The treatment of isolated, symptomatic chondral lesions in the knee remains a challenge, with several surgical options commonly employed including microfracture,^44^ mosaicplasty,^27^ osteoarticular transplantation system, and autologous chondrocyte implantation (ACI).^6^ Traditional ACI techniques required the cultivation and subsequent suspension of chondrocytes under a periosteal flap,^6^ with the second-generation technique employing a biodegradable collagen membrane to contain the cells within the defect.^5^ For this reason, traditional postoperative rehabilitation and weightbearing (WB) protocols were very conservative. However, the current third-generation procedure seeds the cultivated chondrocytes directly onto a type 1/3 collagen membrane, with the membrane fixed to the underlying subchondral bone with fibrin glue (matrix-induced ACI [MACI]). Therefore, this is a potential advantage of third-generation MACI, lending itself to a more accelerated rehabilitation pathway.

The important role of rehabilitation in optimizing outcomes after MACI has been well reported.^10,26,28,37,39,40^ In particular, MACI permits a more accelerated rehabilitation pathway, with respect to the graduated increase in WB capacity and the timing of return to full WB after the procedure, with several studies having now been reported.^13,18,49^ More recently, the 10-year outcomes of a randomized controlled trial were published that reported the benefit on an 8-week (vs 12-week) return to full WB.^14^ Nonetheless, published reports of clinical and radiological outcomes to 10 years after MACI still remain limited, and it is still not known how accelerated these early WB protocols can be. While they cannot be so aggressive that they risk early knee overload and/or jeopardize the integrity of the primitive graft, the lengthy conservative postoperative period often encountered given the need to protect the graft is a hindrance for patients with respect to the return to activities of daily living (ADLs) and work activities.

Therefore, the current study sought to assess the longer term clinical and magnetic resonance imaging (MRI)–based outcome of MACI, as well as investigate any differences in clinical or radiological outcome as a result of a 6-week (vs 8-week) return to full WB after MACI. It was hypothesized that (1) clinical outcomes would improve over the 10-year period, while there would be no differences between WB groups; (2) a high level of patient satisfaction with pain relief, as well as participation in recreational and sporting activities, would be observed at 10 years across all patients; (3) no group differences would be observed in objective measures of lower limb strength and functional hop test symmetry, with adequate (≥90%) mean limb symmetry indices (LSIs) observed at 10 years after surgery; and (4) no significant decline in MRI-based scores would be observed over the duration of the postoperative timeline to 10 years, with no group differences.

## Methods

### Participants

**Figure. 1.**
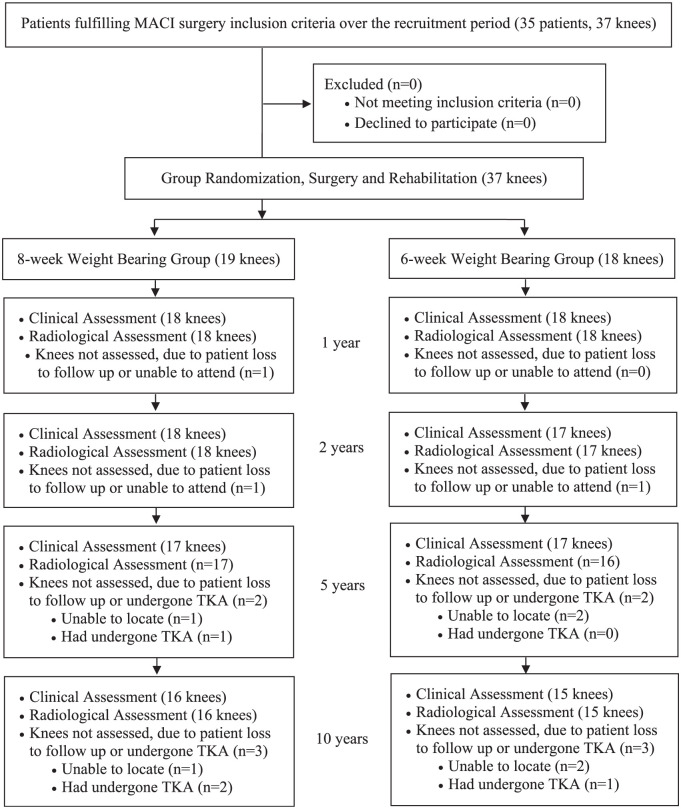
Flowchart demonstrating patient recruitment, as well as the randomization, clinical and radiological assessment of each knee inlcuded, over the 10-year postoperative period.

A total of 35 patients were recruited, with 37 knees (i.e. two patients had both knees enrolled at different timepoints) randomized to a 6-week (n = 18) or 8-week (n = 19) return to full WB after MACI performed in the tibiofemoral joint (Table 1) between January 2010 and April 2014. While the flow of patient recruitment and evaluation over the 10-year postoperative period is outlined in Figure 1, 31 knees (84%; 6 weeks, n = 15; 8 weeks, n = 16) were reviewed clinically at the final 10-year review, with 3 knees (6 weeks, 2; 8 weeks, 1) not assessed due to patient loss to follow-up and 3 knees (6 weeks, 1; 8 weeks, 2) having already had total knee arthroplasty (TKA) performed before 10 years.

**Table 1:** Preoperative Patient Demographics and Injury/Surgery Parameters for each Knee Independently Randomized to one of the two Weightbearing Protocols*^*a*^*

Variable	6 Weeks	8 Weeks
Patients (knees)	17 (18)	18 (19)
Sex, male/female	9/9	12/7
Age, y	36.4 (21.0-55.0)	36.4 (23.0-53.0)
Height, m	1.76 (1.55-2.03)	1.77 (1.54-1.92)
Weight, kg	82.3 (46.0-130.0)	79.4 (46.0-109.3)
Body mass index	26.2 (18.4-32.1)	25.2 (19.1-33.1)
Defect location, MFC/LFC	13/5	14/5
Previous procedures	1.1 (0-4)	1.0 (0-4)
Duration of symptoms, y	7.5 (1-25)	6.8 (1-25)
Defect size, cm^2^	3.15 (1.00-6.25)	2.89 (1.00-7.70)
≤2.0	7	8
2.1-3.0	4	5
3.1-4.0	4	2
4.1-5.0	1	2
≥5.1	2	2

a Data are presented as mean (range) or n. LFC, lateral femoral condyle; MFC, medial femoral condyle.

All patients had pain and symptoms associated with grade 3 or 4 chondral lesions that were initially assessed via baseline MRI with the International Cartilage Regeneration & Joint Preservation Society (ICRS) chondral defect classification system.^8^ While the first-stage arthroscopic cartilage biopsy was used to confirm the ICRS grading, no patients had a better ICRS grading, albeit 3 patients had an arthroscopic ICRS grading that was worse than the preliminary MRI-based evaluation. Trial inclusion also required an age range of 15 to 65 years and a body mass index of <35. Patients with ligamentous instability, varus/valgus abnormalities (>5° tibiofemoral anatomic angle), and those with ongoing progressive inflammatory arthritis were excluded. Ethics approval for this study was obtained from the Hollywood Private Hospital, and all patients provided their written informed consent to participate.

### The MACI Surgical Technique

The MACI surgical technique has been previously described.^13^ Initially, in a first-stage arthroscopic procedure a small sample of articular cartilage was harvested from a non-WB area of the knee. After chondral harvest, chondrocytes were isolated, cultured, and seeded onto a type 1/3 collagen membrane (ACI-Maix; Matricel GmbH). In a second surgery via an open arthrotomy, the cartilage defect was prepared by removing all damaged cartilage down to, but not through, the subchondral bone. The seeded membrane was cut to fit the defect and glued to the underlying bone with fibrin glue.

### Postoperative Rehabilitation

The early in-patient management pathway was standardized for all patients, irrespective of group allocation, and included cryotherapy and training on proficient toe-touch ambulation with crutches, continuous passive motion (0°-30°), active ankle movement to encourage lower extremity circulation, and isometric quadriceps, hamstrings, and gluteal contractions. After hospital discharge, all patients attended the same outpatient rehabilitation clinic and participated in a supervised outpatient rehabilitation program consisting of 2 supervised sessions per week over a 12-week period, with ongoing advice and education provided as required up until 12 months. This included a graduated increase in knee range of motion and progressive exercises prescribed to improve lower limb and trunk strengthening and functional WB capacity, with eventual progression toward work-, activity-, and sport-related tasks.^13^ This program was standardized, although advancement throughout the program was further dictated by other individual variables such as graft size, concomitant surgeries, and the individual patient's conditioning and tolerance to exercise.

**Figure 2.**
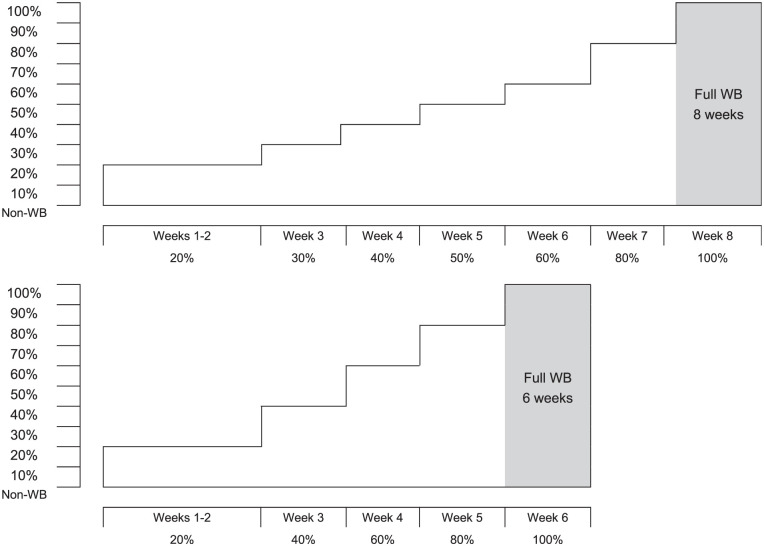
The weightbearing (WB) gradients that were followed by matrix-induced autologous chondrocyte implantation patients randomized to the 6-week and 8-week rehabilitation groups.

While the aforementioned management pathway was standardized across all patients, the 2 groups underwent a varied postoperative WB gradient with the time to attain full unaided WB different between groups. While these WB protocols have been previously published,^13^ they are also shown in Figure 2. The bathroom scale method was employed to teach WB restrictions,^11,26^ and previous research has demonstrated improved accuracy with more practice.^11^ This was an important part of every rehabilitation session and, while compliance with the WB levels could not be assessed external to the clinic, patients were well-educated on the importance of maintaining their WB capacity to the best of their ability.

### Clinical Assessment

First, several patient-reported outcome measures (PROMs) were collected presurgery, as well as 1, 2, 5, and 10 years after surgery. These consisted of the Knee injury and Osteoarthritis Outcome Score (KOOS),^42^ which included 5 subscales: knee Pain, Symptoms, ADLs, Sport and Recreation, and knee-related Quality of Life; the Lysholm Knee Scale,^33^ the Tegner Activity Scale (TAS),^45^ a visual analog scale (VAS) for pain to assess the frequency and severity of knee pain (0-10, with 0 being no pain and 10 being worst pain imaginable), and the 36-item Short Form Health Survey (SF-36),^3^ including a mental (MCS) and physical component summary. Specifically at 10 years, a patient satisfaction questionnaire was employed to evaluate satisfaction with the surgery to relieve pain, improve the ability to perform normal daily and work activities, improve the ability to return to recreational activities (including walking, swimming, cycling, golf, dancing), and improve the ability to participate in sport (including sports such as tennis, netball, soccer, and football). A Likert response scale was employed with descriptors *very satisfied*, *somewhat satisfied*, *somewhat dissatisfied*, and *very dissatisfied*.

Second, a range of objective assessments were undertaken at all postoperative time points (1, 2, 5, and 10 years). These were assessed on both the operated and the nonoperated limbs and included active knee flexion and extension range as well as peak isokinetic knee extensor (quadriceps) and flexor (hamstrings) torque. Active knee range was assessed using a long-arm goniometer with the patient in supine. Peak knee extensor and flexor torque was assessed using an isokinetic dynamometer (Isosport International) at an angular velocity of 90 deg/s. Specifically at 10 years after surgery, the single horizontal (SHD), single lateral (LHD) and single medial (MHD) hop tests for distance were administered. All clinical measurements were undertaken within the same private outpatient therapy clinic, by a qualified, and experienced and independent therapist.

### MRI Assessment

High-resolution MRI using a 1.5- or 3-T Siemens Symphony scanner was used to assess graft status at 1, 2, 5, and 10 years after surgery. Standardized proton density and T2-weighted fat-saturated images were obtained in coronal and sagittal planes (slice thickness, 3 mm; field of view, 14-15 cm; and 512 matrix in ≥1 axis for proton density images with a minimum 256 matrix in 1 axis for T2-weighted images). Axial proton density fat-saturated images were obtained (slice thickness, 3-4 mm; field of view, 14-15 cm; minimum 224 matrix in ≥1 axis).

Pertinent parameters of graft repair (graft infill, signal intensity, border integration, surface contour, tissue structure, effusion, subchondral lamina, and bone) were assessed on MRI as per the MOCART (magnetic resonance observation of cartilage repair tissue) scoring system.^34,41,46,48^ Each graft parameter was scored from 1 to 4 (1 = poor; 2 = fair; 3 = good; 4 = excellent) in comparison with the native cartilage (with an additional score of 3.5 provided for graft hypertrophy in the category of graft infill).^35,46^ Furthermore, an MRI composite score was also calculated by multiplying each individual score by a weighting factor^41^ and adding the scores together.^15^ MRI evaluation was performed by an independent, experienced musculoskeletal radiologist, blinded to group randomization (S.K.). For the current study, graft failure was defined by an exposed subchondral bone bed with no evidence of repair tissue on MRI, or the patient having undergone conversion to TKA within the designated follow-up period (although it should be appreciated that conversion to TKA is not a direct reflection of graft status, rather more of extensive knee joint degeneration).

### Statistical Analysis

A priori power calculation was performed using G-Power before study onset for the primary outcome variable (the KOOS Pain subscale), demonstrating that 28 knees (14 in each group) were required to reveal differences at the 5% significance level, with 90% power and employing a large effect size (1.1) as reported by previous research.^50^

First, LSIs were calculated for the isokinetic knee extensor and flexor torque assessments, as well as the single-limb hop tests (representing a score of the operated limb as a percentage of the nonoperated limb). Normality of distribution of continuous data was assessed and confirmed via the Shapiro-Wilk test. Subsequently, the means and standard deviation of all PROMs, objective measures (active knee flexion and extension range on the operated and nonoperated limbs, as well as LSIs for strength and hop measures), and MRI-based scores were presented, with repeated measures analysis of variance used to investigate the change in measures over time and between groups. Where a significant group or interaction effect was found, post hoc independent *t* tests were used to determine time points at which the 2 groups differed. Where appropriate, *t* tests were also used to evaluate changes specifically between 5 and 10 years after surgery. For the 2 WB groups, the number (and percentage) of patients reporting very satisfied, somewhat satisfied, somewhat dissatisfied, and very dissatisfied within each of the satisfaction domains at 10 years was presented. The number (and percentage) of grafts evaluated as good or excellent and poor or fair as per the MOCART MRI-based scoring system for each of the 8 variables, for each of the 2 groups, was presented at 10 years after surgery. Where appropriate, statistical analysis was performed using SPSS software (Version 28.0; SPSS Inc). Statistical significance was determined at *P* < .05.

## Results

All PROMs significantly improved (*P* < .0001) over the pre- and postoperative period to 10 years after surgery, apart for the SF-36 MCS (*P* = .57) (Table 2). There were no group differences (*P* > .05) observed (Table 2). Furthermore, *t* tests indicated that there were no significant changes (*P* > .05) in any PROM or objective measure (knee range and strength LSIs) from 5 to 10 years after surgery. Overall, satisfaction with pain relief of 93.3% and 87.5% was reported in the 6-week and 8-week WB groups, respectively (Table 3). Furthermore, satisfaction with sports particpation of 86.7% and 81.3% was reported in the 6-week and 8-week WB groups, respectively (Table 3). The peak knee extensor strength LSI significantly increased from 1 to 10 years after surgery (Table 4), with no changes in the peak knee flexor strength LSI (Table 4). While no group differences (*P* > .05) were observed for any of the objective measures, mean LSIs for both groups across all measures (knee extensor and flexor torque and the SHD, LHD, and MHD) at 10 years ranged from 99.1% to 103.8% (Table 4).

**Table 2:** Patient-Reported Outcome Measures (PROMs) Presurgery and at 1, 2, 5, and 10 Years After Surgery for Both Weightbearing Groups*^*a*^*

Time Point	Group	KOOS (Pain)	KOOS (Symptoms)	KOOS (ADL)	KOOS (Sport/Rec)	KOOS (QOL)	LKS	TAS	SF-36 (PCS)	SF-36 (MCS)	VAS-F	VAS-S
Presurgery	6 weeks	63.2 (13.2)	64.0 (17.0)	73.8 (15.9)	26.8 (16.7)	31.7 (14.7)	60.3 (18.1)	2.8 (1.1)	35.1 (9.0)	51.1 (9.3)	6.3 (2.4)	5.7 (1.9)
	8 weeks	66.9 (9.3)	73.1 (11.2)	80.6 (9.4)	36.2 (25.1)	35.6 (11.4)	56.7 (19.6)	3.0 (0.8)	39.6 (5.8)	51.5 (10.2)	6.5 (2.2)	5.3 (2.4)
1 y	6 weeks	86.8 (6.6)	85.8 (9.7)	92.5 (5.9)	60.7 (19.6)	70.8 (9.6)	85.1 (9.6)	5.3 (0.9)	46.7 (7.8)	59.3 (5.6)	1.6 (0.7)	2.0 (1.2)
	8 weeks	88.3 (6.3)	88.7 (8.5)	95.4 (5.1)	64.8 (24.1)	59.1 (16.1)	86.8 (8.8)	5.8 (1.6)	49.6 (5.5)	57.1 (5.1)	1.3 (1.0)	1.8 (1.5)
2 y	6 weeks	88.0 (13.5)	86.1 (10.6)	92.3 (9.2)	69.0 (24.7)	71.5 (19.0)	87.2 (9.8)	5.3 (1.1)	49.1 (8.5)	54.9 (6.5)	1.6 (2.1)	1.7 (1.6)
	8 weeks	90.2 (10.6)	88.8 (7.7)	95.4 (9.1)	69.4 (25.1)	67.3 (22.9)	87.7 (8.0)	5.8 (1.6)	50.2 (8.6)	58.0 (4.3)	1.4 (1.6)	1.8 (1.6)
5 y	6 weeks	87.4 (19.7)	83.5 (18.9)	93.4 (17.7)	80 (29.8)	73.1 (30.0)	82.6 (17.7)	5.3 (2.1)	50.3 (11.6)	55.6 (7.1)	1.9 (2.9)	1.8 (2.2)
	8 weeks	90.1 (10.8)	88.4 (9.3)	91.7 (11.4)	75.6 (26.6)	70.3 (24.2)	79.9 (19.9)	5.2 (1.6)	52.6 (4.7)	55.1 (9.3)	1.9 (1.9)	1.9 (2.3)
10 y	6 weeks	88.4 (9.1)	87.6 (9.9)	95.0 (7.1)	78.0 (20.9)	69.4 (19.2)	83.1 (12.5)	5.0 (1.8)	48.8 (7.8)	50.7 (9.1)	1.5 (1.2)	1.6 (1.1)
	8 weeks	88.1 (9.9)	89.6 (8.2)	93.4 (9.2)	76.8 (20.1)	69.3 (18.3)	85.5 (12.1)	5.1 (1.5)	51.7 (5.9)	52.4 (9.5)	1.9 (1.9)	1.9 (1.4)
Time effect, *P*		**<.0001**	**<.0001**	**<.0001**	**<.0001**	**<.0001**	**<.0001**	**<.0001**	**<.0001**	.57	**<.0001**	**<.0001**
Group effect, *P*		.54	.69	.56	.97	.47	.54	.66	.83	.99	.25	.21
Interaction effect, *P*		.82	.96	.72	.28	.60	.93	.21	.98	.68	.81	.52

a Data are presented as mean (SD), with analysis of variance results. Bold *P* values indicate statistically significant. ADL, Activities of Daily Living; KOOS, Knee injury and Osteoarthritis Outcome Score; LKS, Lysholm Knee Score; MCS, Mental Component Summary; PCS, Physical Component Summary; QOL, Quality of Life; SF-36, 36-item Short Form Health Survey; Sport/Rec, Sport and Recreation; TAS, Tegner Activity Score; VAS-F, visual analog scale for pain–frequency; VAS-S, visual analog scale for pain–severity.

**Table 3:** Satisfaction Gradings for Each of the Satisfaction Items for the 2 Weightbearing Groups 10 Years Postoperatively*^*a*^*

Group	Satisfaction Item	Pain Relief	Improving Ability to Undertake ADLs	Improving Ability to Participate in Recreational Activities	Improving Ability to Participate in Sport	Overall Satisfaction
6 weeks (n = 15)	Very satisfied	10	12	10	9	10
	Satisfied	4	2	4	4	4
	Dissatisfied	1	1	1	2	1
	Very dissatisfied	0	0	0	0	0
	Satisfied overall	14 (93.3)	14 (93.3)	14 (93.3)	13 (86.7)	14 (93.3)
8 weeks (n = 16)	Very satisfied	9	10	9	7	9
	Satisfied	5	4	5	6	5
	Dissatisfied	2	2	2	2	2
	Very dissatisfied	0	0	0	1	0
	Satisfied overall	14 (87.5)	14 (87.5)	14 (87.5)	13 (81.3)	14 (87.5)

a Data are presented as n or n (%). Satisfaction gradings include very satisfied, somewhat satisfied, somewhat dissatisfied, and very dissatisfied. ADL, Activities of Daily Living.

**Table 4:** Active Knee Range of Motion and physical performance test LSIs*^*a*^*

Time Point	Variable	Knee Flexion, deg		Knee Extension, deg		Knee Extensor Strength LSI	Knee Flexor Strength LSI	SHD LSI	LHD LSI	MHD LSI
		Operated	Non-operated	Operated	Non-operated					
1 y	6 weeks	140.9 (6.7)	142.4 (6.8)	–1.2 (1.8)	–2.1 (1.9)	82.1 (14.9)	107.8 (14.1)	N/A	N/A	N/A
	8 weeks	144.9 (5.6)	144.7 (7.0)	–1.6 (1.8)	–2.6 (1.9)	83.5 (17.9)	108.8 (16.1)			
2 y	6 weeks	143.6 (7.5)	142.4 (7.5)	–1.8 (1.6)	–1.9 (1.7)	93.7 (14.7)	101.1 (8.5)			
	8 weeks	144.8 (6.3)	144.4 (7.1)	–2.0 (1.8)	–2.3 (1.9)	87.5 (11.8)	101.2 (9.1)			
5 y	6 weeks	144.5 (8.7)	144.9 (9.0)	–0.8 (1.4)	–0.9 (1.6)	99.8 (12.4)	104.5 (9.0)			
	8 weeks	147.5 (6.4)	147.6 (6.3)	–0.7 (1.9)	–1.0 (1.7)	97.8 (12.4)	105.1 (10.5)			
10 y	6 weeks	146.5 (8.9)	148.3 (7.7)	–0.8 (1.5)	–0.8 (1.5)	100.8 (12.1)	101.9 (14.0)	100.5 (6.9)	103.8 (6.6)	99.0 (3.7)
	8 weeks	149.3 (5.7)	147.7 (8.4)	–0.6 (0.9)	–0.9 (1.0)	99.1 (8.6)	100.1 (8.7)	99.1 (4.0)	99.7 (10.1)	99.7 (8.4)
Time effect, *P*		**.019**	**.012**	**<.0001**	**<.0001**	**<.0001**	.357	N/A	N/A	N/A
Group effect, *P*		.61	.95	.61	.52	.54	.61	.61	.09	.87
Interaction effect, *P*		.78	.52	.14	.13	.54	.39	N/A	N/A	N/A

a Data are presented as mean (SD), with analysis of variance results. Bold *P* values indicate statistically significant. LSI, limb symmetry index; SHD, single horizontal hop for distance; LHD, single lateral hop for distance; MHD, single medial hop for distance; N/A, not available.

**Figure 3.**
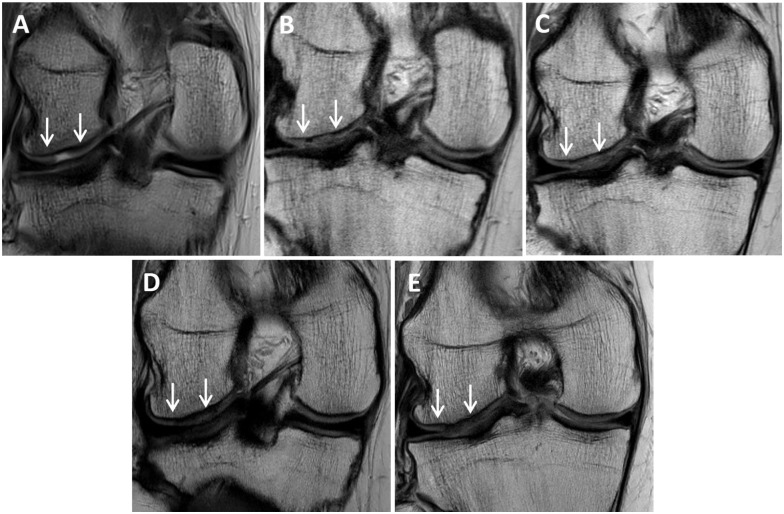
Proton density fast spin-echo magnetic resonance images of (A) a chondral defect preoperatively on the lateral femoral condyle (between white arrows), as well as the subsequent matrix-induced autologous chondrocyte implantation graft at (B) 1 year, (C) 2 years, (D) 5 years, and (E) 10 years.

No significant change (*P* > .05) was observed for any graft parameter, nor the MRI composite score, from 1 to 10 years after surgery (Table 5). Furthermore, while a significant group effect (*P* = .028) was observed for signal intensity, indicating tissue signal more like the native articular cartilage in the 6-week WB group, no other group differences were observed (Table 5). MRI images of a patient in the series who underwent MACI on the lateral femoral condyle are shown in Figure 3.

**Table 5:** MRI-Based Graft Parameter Scores and MRI Composite Score for Both Weightbearing Groups at 1, 2, 5, and 10 Years After Surgery*^*a*^*

Time Point	Group	Graft Infill	Signal Intensity	Border Integration	Surface Contour	Structure	Subchondral Lamina	Subchondral Bone	Effusion	MRI Composite Score
1 year	6 weeks	3.53 (0.54)	3.00 (0.96)	3.24 (0.83)	3.18 (1.21)	3.24 (0.83)	3.47 (0.62)	3.41 (0.70)	3.71 (0.57)	3.32 (0.58)
	8 weeks	3.18 (0.95)	2.74 (0.83)	2.79 (1.16)	2.63 (1.08)	3.16 (1.06)	3.31 (0.72)	3.00 (0.99)	3.95 (0.25)	3.01 (0.75)
2 years	6 weeks	3.47 (0.44)	3.30 (0.58)	3.29 (0.58)	3.65 (0.47)	3.58 (0.65)	3.59 (0.52)	3.35 (0.80)	3.88 (0.36)	3.46 (0.27)
	8 weeks	3.18 (0.89)	2.58 (0.81)	2.79 (0.85)	2.79 (0.92)	3.16 (0.89)	3.42 (0.53)	3.05 (0.98)	3.95 (0.30)	3.00 (0.78)
5 years	6 weeks	3.41 (0.34)	3.15 (0.29)	2.99 (1.14)	3.11 (1.15)	3.00 (1.05)	3.15 (0.58)	3.10 (1.16)	3.77 (0.45)	3.19 (0.40)
	8 weeks	3.33 (0.45)	2.82 (0.15)	2.89 (1.41)	2.97 (1.51)	3.14 (1.21)	3.51 (0.48)	3.00 (1.41)	3.71 (0.49)	3.07 (0.60)
10 years	6 weeks	3.12 (0.62)	3.33 (0.50)	2.61 (1.00)	3.05 (1.12)	2.85 (0.93)	3.00 (0.60)	2.87 (1.30)	3.83 (0.39)	3.06 (0.38)
	8 weeks	3.02 (0.58)	3.00 (0.12)	2.63 (1.32)	2.73 (1.36)	2.55 (1.41)	3.35 (0.53)	3.00 (1.31)	3.75 (0.46)	2.88 (0.47)
Time effect, *P*		.11	.22	.10	.23	.38	.88	.09	.56	.17
Group effect, *P*		.95	**.028**	.83	.10	.81	.21	.89	.74	.44
Interaction effect, *P*		.42	.70	.87	.56	.73	.12	.13	.16	.72

a Data are presented as mean (SD), with analysis of variance results. Bold *P* value indicates statistically significant. MRI, magnetic resonance imaging.

**Figure 4.**
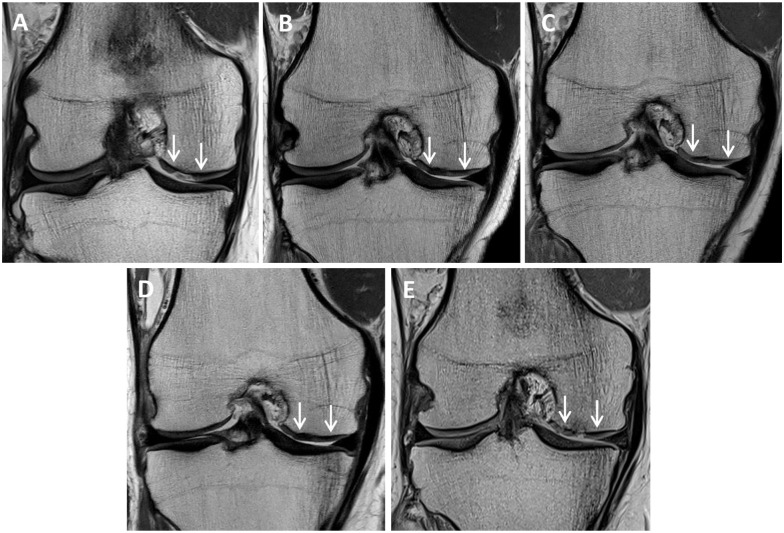
Proton density fast spin-echo magnetic resonance images of (A) a chondral defect preoperatively on the medial femoral condyle (between white arrows), as well as the subsequent matrix-induced autologous chondrocyte implantation graft at (B) 1 year, (C) 2 years, and (D) 5 years, with graft failure demonstrated at (E) 10 years.

**Figure 5.**
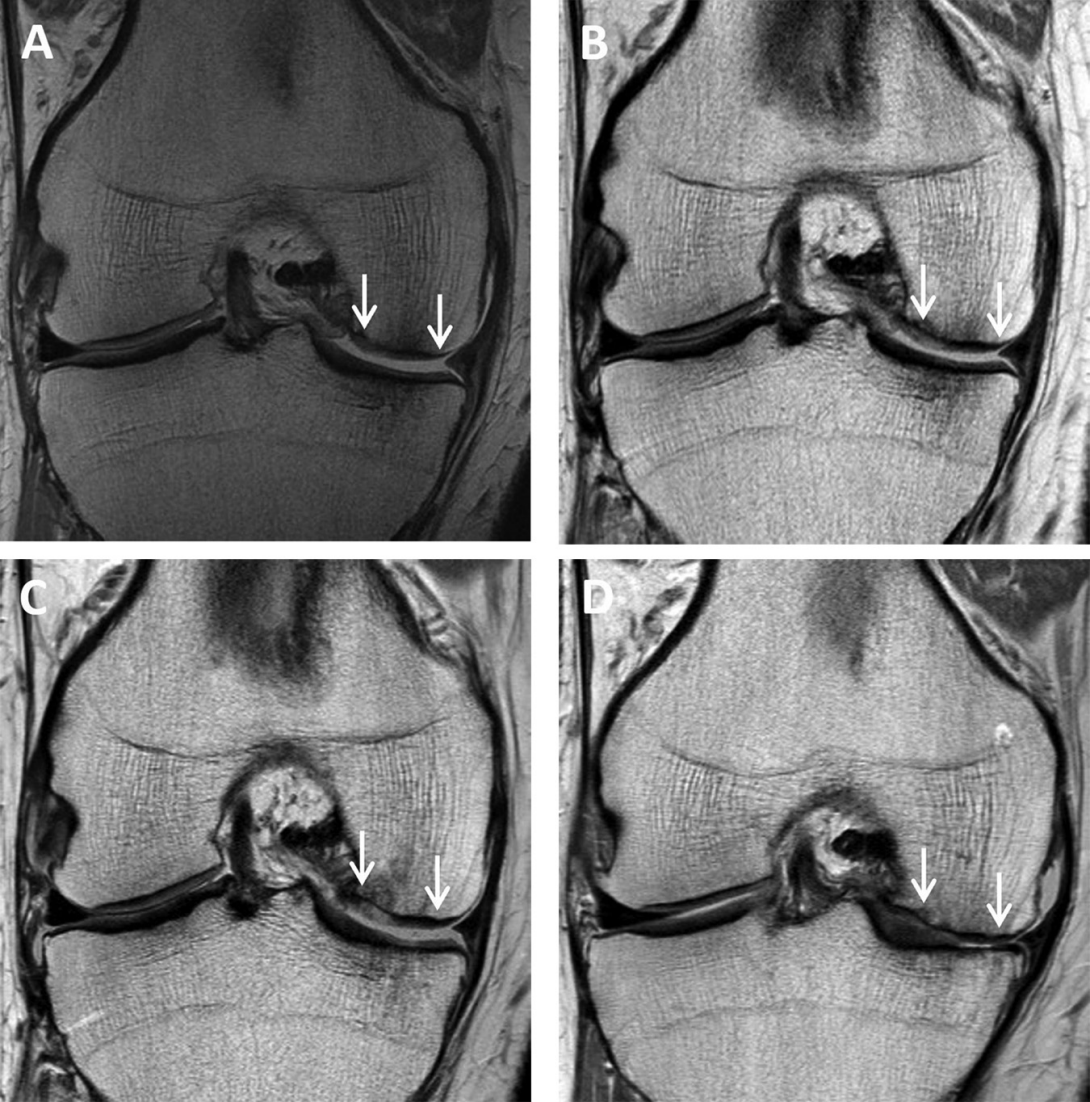
Proton density fast spin-echo magnetic resonance images of a matrix-induced autologous chondrocyte implantation (MACI) graft (between white arrows) on the medial femoral condyle at (A) 3 months, (B) 1 year, (C) 2 years, and (D) 5 years, with 5-year imaging reflecting a failed graft and this patient subsequently undergoing knee replacement surgery at 8 years after the primary MACI surgery.

Of the 31 grafts (84%) that were assessed via MRI at 10 years, 1 graft had failed (in the 8-week group). MRI images of this patient presurgery and at 1, 2, 5, and 10 years after surgery are shown in Figure 4. However, of the 6 knees that were not clinically assessed at 10 years (including n = 3 knees that could not be assessed due to patients loss to follow-up) (Figure 1), and 3 knees patients (6 weeks, n = 1; 8 weeks, n = 2) that had already undergone TKA before 10 years. Therefore, an overall failure rate at 10 years of 11.8% was observed. MRI images at 3 months and 1, 2, and 5 years for one of the patients who underwent TKA at 8 years after his MACI are shown in Figure 5.

## Discussion

The current study has demonstrated sound and sustained clinical and MRI-based improvement after MACI to 10 years after surgery, with high levels of patient satisfaction. Furthermore, no differences in mid- or long-term clinical outcomes were observed in patients following a 6-week (versus 8-week) WB pathway after undergoing MACI on the tibiofemoral joint, suggesting the 6-week return to unaided full WB gait is safe.

Most clinical outcomes significantly improved over the postoperative period (all PROMs apart from the SF-36 MCS, knee flexion and extension range on the operated and nonoperated limbs, and the knee extensor strength LSI). Further to this, there were no differences observed between the 2 WB groups, with >80% of each group at 10-year follow-up reporting being satisfied (or very satisfied) across each of the satisfaction domains (pain relief, improving their ability to perform ADLs, and participation in recreational and sporting activities). This was largely in support of the first 2 hypotheses. Previous research has reported encouraging clinical outcomes to 5 years after MACI,^4,7,16,18,21,23,31,49^ with some more recent studies now reporting encouraging outcomes to 10 years and beyond.^1,14,17,19,22,38^ Further to this, one of these studies reported the beneficial effects of an 8-week (versus 12-week) return to full WB gait after MACI,^14^ with 10-year clinical scores similar to those observed in the current randomized controlled trial.

While the knee extensor strength LSI improved over time, which may also highlight the delayed and longer term recovery required for quadriceps strength after a procedure like MACI, no group differences were observed in the knee extensor, knee flexor, or hop test LSIs at 10 years. Furthermore, mean LSIs for these objective measures of strength and function at 10 years were high and all well above the hypothesized 90%, in support of the third hypothesis. While we lack any evidence as to the association between lower physical performance LSIs and any risk of subsequent knee injury after MACI, some studies have reported an increased risk of rerupture in patients after anterior cruciate ligament (ACL) reconstruction if returning to pivoting sports without meeting 90% LSI criteria in strength- and hop-based assessments.^25,32^ We do acknowledge some potential limitations associated with employing LSIs to present strength and hop outcomes. In ACL patients, this includes the variation in LSI “cutoff” values that have been employed to report satisfactory recovery^2,12,25,30,43^ and their potential for overestimating knee function^47^ when also considering the presence of bilateral neuromuscular deficits.^9,24,29^ Nonetheless, given the variability in the cohort that underwent MACI—which may differ from an often younger ACL cohort—we believe the LSIs reported in the current study are more than adequate and the high mean LSIs observed at 10 years after surgery in the current study would suggest an adequate level of recovery in strength and functional symmetry.

While mean scores for each of the graft-related parameters (graft infill, signal intensity, border integration, surface contour, tissue structure, effusion, subchondral lamina, and bone), as well as the overall MRI composite score, were all above 2.5 at 10 years (with each of the parameters scored from 1 to 4 for each patient), it was encouraging that no parameter demonstrated a statistically significant decline over the 10-year postoperative period. Despite the failures observed over the 10-year period (including 1 graft failure identified on MRI at 10 years, along with 3 prior patients who had already proceeded toward TKA because of graft failure and persistent/recurrent symptoms), a sound level of graft sustainability was otherwise seen that was in support of the fourth hypothesis. Of interest, mean scores for the MRI-based signal intensity parameter were consistently (and significantly) higher for the 6-week WB group, while 3 (of the 4) failures were observed in the 8-week group. Therefore, while no other group-based differences in MRI scores were observed, the 6-week group was certainly not inferior. The MRI-based scores observed do appear consistent with more recent 10-year studies reporting outcomes after MACI.^14,17,19^

The important role of rehabilitation as a supplement to the MACI surgical procedure, along with the important constituents of such a progressive program, has been well-established.^10,20,26,28,37,39,40^ Pertinent to the current study, this includes the progressive increase toward full and unaided WB gait. Traditionally, the return to full WB after ACI has been conservative,^36^ often related to early ACI generations and the need to suture a periosteal (first generation) or collagen (second generation) membrane around the lesion to contain the cells suspended in a solution. Theoretically, a postoperative pathway that is too aggressive could therefore increase the risk of early graft delamination, albeit the natural clinical side effect of a more protective WB and activity progression results in increased patient frustration, a delayed return to various lifestyle and work-based activities, and an increase in muscle deterioration. Since that time and, specific to third-generation ACI, studies have reported the benefit of a return to full WB by 8 weeks after surgery.^13,14,18,49,50^ The outcomes of the current study would suggest that the accelerated 6-week (versus 8-week) WB pathway produced at least comparable outcomes, providing rationale for the faster return to full WB gait. While it may be argued that the additional 2 weeks using an ambulatory aid is minimal, this can be of a larger clinical relevance to the patient who is seeking a quicker return to full and unaided WB gait, while this often has more significant ramifications for returning to other lifestyle- and work-based activities.

### Limitations

Some limitations should be acknowledged in the current study. First, despite the encouraging longer term outcomes across both WB groups and the study recruitment attaining the desired sample size (which was dictated by the primary outcome variable, the KOOS Pain subscale), we acknowledge the relatively small sample size overall. This may mean that other variables were underpowered and may limit study conclusions. Second, while the current study sought to investigate longer term outcomes associated with MACI and investigate the effect of 2 different WB programs, a control or comparator group undergoing nonoperative management or a different cartilage repair procedure was not employed. While patients in the 6- and 8-week WB groups reported a mean duration of symptoms of 7.5 and 6.8 years, respectively (suggesting many had already failed nonoperative management), the preferred treatment option for these symptomatic cartilage defects at the time of the study was MACI. This made a comparative study difficult. Third, while a history of previous surgical procedures was collected and documented (with no differences between the WB groups), an accurate history of other adjunctive treatments (such as corticosteroid or hyaluronic acid injections, or a detailed history of rehabilitation before surgery) was not collected and could not be compared between groups. Fourth, while the time to attain full WB could be easily and accurately achieved by removal of patient crutches, ensuring the specific gradient of progressive WB up until the time of full WB was more challenging. WB training was an important part of every supervised session, while patients were educated on the importance of adhering to WB protocols. However, WB compliance and the degree of WB borne during gait were not measured external to the clinic setting. Finally, the study employed several PROMs, including the TAS to obtain an assessment of postoperative activity level, but did not seek to obtain a more accurate measure of actual return to sport, timing of return, and level of sporting activity. Furthermore, there are a range of other higher level objective means of assessment, including other hop tests. A battery of testing measures designed to assess a higher level of functional capacity may be employed in future research.

## Conclusion

The current study demonstrated that MACI produced sustained clinical and MRI-based improvement over 10 years, with high levels of patient satisfaction. Furthermore, no differences in clinical or radiological outcomes were observed in patients following a 6-week (vs 8-week) return to full WB after MACI on the tibiofemoral joint. Therefore, while patient follow-up will continue to assess the longevity of these grafts, the outcomes suggest that the accelerated 6-week return to unaided full WB gait is safe and can be undertaken in a controlled environment without fear of jeopardizing the early integrity of the graft.

## References

[1] Aldrian S Zak L Wondrasch B , et al. Clinical and radiological long-term outcomes after matrix-induced autologous chondrocyte transplantation: a prospective follow-up at a minimum of 10 years. Am J Sports Med. 2014;42(11):2680-2688. 25204296 10.1177/0363546514548160

[2] Barfod KW Feller JA Hartwig T Devitt BM Webster KE. Knee extensor strength and hop test performance following anterior cruciate ligament reconstruction. Knee. 2019;26(1):149-154. 30554909 10.1016/j.knee.2018.11.004

[3] Bartlett W Gooding CR Carrington RWJ Briggs TWR Skinner JA Bentley G. The role of the Short Form 36 Health Survey in autologous chondrocyte implantation. Knee. 2005;12(4):281-285. 16005633 10.1016/j.knee.2004.11.001

[4] Behrens P Bitter T Kurz B Russlies M. Matrix-associated autologous chondrocyte transplantation/implantation (MACT/MACI)—5-year follow-up. Knee. 2006;13(3):194-202. 16632362 10.1016/j.knee.2006.02.012

[5] Bentley G Biant LC Carrington RW , et al. A prospective, randomised comparison of autologous chondrocyte implantation versus mosaicplasty for osteochondral defects in the knee. J Bone Joint Surg Br. 2003;85(2):223-230. 12678357 10.1302/0301-620x.85b2.13543

[6] Brittberg M Lindahl A Nilsson A Ohlsson C Isaksson O Peterson L. Treatment of deep cartilage defects in the knee with autologous chondrocyte transplantation. N Engl J Med. 1994;331(14):889-895. 8078550 10.1056/NEJM199410063311401

[7] Brittberg M Recker D Ilgenfritz J Saris DBF Group SES . Matrix-applied characterized autologous cultured chondrocytes versus microfracture: five-year follow-up of a prospective randomized trial. Am J Sports Med. 2018;46(6):1343-1351. 29565642 10.1177/0363546518756976

[8] Brittberg M Winalski CS. Evaluation of cartilage injuries and repair. J Bone Joint Surg Am. 2003;85-A(suppl 2):58-69. 10.2106/00004623-200300002-00008 12721346

[9] Culvenor AG Alexander BC Clark RA , et al. Dynamic single-leg postural control is impaired bilaterally following anterior cruciate ligament reconstruction: implications for reinjury risk. J Orthop Sports Phys Ther. 2016;46(5):357-364. 26999412 10.2519/jospt.2016.6305

[10] Deszczynski J Slynarski K. Rehabilitation after cell transplantation for cartilage defects. Transplant Proc. 2006;38(1):314-315. 16504734 10.1016/j.transproceed.2005.12.074

[11] Ebert JR Ackland TR Lloyd DG Wood DJ. Accuracy of partial weight bearing after autologous chondrocyte implantation. Arch Phys Med Rehabil. 2008;89(8):1528-1534. 18674988 10.1016/j.apmr.2008.02.019

[12] Ebert JR Edwards P Yi L , et al. Strength and functional symmetry is associated with post-operative rehabilitation in patients following anterior cruciate ligament reconstruction. Knee Surg Sports Traumatol Arthrosc. 2018;26(8):2353-2361. 28916871 10.1007/s00167-017-4712-6

[13] Ebert JR Edwards PK Fallon M Ackland TR Janes GC Wood DJ. Two-year outcomes of a randomized trial investigating a 6-week return to full weightbearing after matrix-induced autologous chondrocyte implantation. Am J Sports Med. 2017;45(4):838-848. 27881381 10.1177/0363546516673837

[14] Ebert JR Fallon M Ackland TR Janes GC Wood DJ. Minimum 10-year clinical and radiological outcomes of a randomized controlled trial evaluating 2 different approaches to full weightbearing after matrix-induced autologous chondrocyte implantation. Am J Sports Med. 2020;48(1):133-142. 31765228 10.1177/0363546519886548

[15] Ebert JR Fallon M Robertson WB , et al. Radiological assessment of accelerated versus traditional approaches to post-operative rehabilitation following matrix-induced autologous chondrocyte implantation (MACI). Cartilage. 2011;2(1):60-72. 26069570 10.1177/1947603510380902 PMC4300786

[16] Ebert JR Fallon M Wood DJ Janes GC. A prospective clinical and radiological evaluation at 5 years after arthroscopic matrix-induced autologous chondrocyte implantation. Am J Sports Med. 2017;45(1):59-69. 27587741 10.1177/0363546516663493

[17] Ebert JR Fallon M Wood DJ Janes GC. Long-term prospective clinical and magnetic resonance imaging-based evaluation of matrix-induced autologous chondrocyte implantation. Am J Sports Med. 2021;49(3):579-587. 33411565 10.1177/0363546520980109

[18] Ebert JR Fallon M Zheng MH Wood DJ Ackland TR. A randomized trial comparing accelerated and traditional approaches to postoperative weightbearing rehabilitation after matrix-induced autologous chondrocyte implantation: findings at 5 years. Am J Sports Med. 2012;40(7):1527-1537. 22539536 10.1177/0363546512445167

[19] Ebert JR Zheng M Fallon M Wood DJ Janes GC. 10-year prospective clinical and radiological evaluation after matrix-induced autologous chondrocyte implantation and comparison of tibiofemoral and patellofemoral graft outcomes. Am J Sports Med. 2024;52(4):977-986. 38384192 10.1177/03635465241227969 PMC10943616

[20] Edwards PK Ackland T Ebert JR. Clinical rehabilitation guidelines for matrix-induced autologous chondrocyte implantation on the tibiofemoral joint. J Orthop Sports Phys Ther. 2014;44(2):102-119. 24175609 10.2519/jospt.2014.5055

[21] Genovese E Ronga M Angeretti MG , et al. Matrix-induced autologous chondrocyte implantation of the knee: mid-term and long-term follow-up by MR arthrography. Skeletal Radiol. 2011;40(1):47-56. 20446086 10.1007/s00256-010-0939-8

[22] Gille J Behrens P Schulz AP Oheim R Kienast B. Matrix-associated autologous chondrocyte implantation: a clinical follow-up at 15 years. Cartilage. 2016;7(4):309-315. 27688839 10.1177/1947603516638901 PMC5029570

[23] Gobbi A Kon E Berruto M , et al. Patellofemoral full-thickness chondral defects treated with second-generation autologous chondrocyte implantation: results at 5 years' follow-up. Am J Sports Med. 2009;37(6):1083-1092. 19465733 10.1177/0363546509331419

[24] Gokeler A Welling W Benjaminse A Lemmink K Seil R Zaffagnini S. A critical analysis of limb symmetry indices of hop tests in athletes after anterior cruciate ligament reconstruction: a case control study. Orthop Traumatol Surg Res. 2017;103(6):947-951. 28428033 10.1016/j.otsr.2017.02.015

[25] Grindem H Snyder-Mackler L Moksnes H Engebretsen L Risberg MA. Simple decision rules can reduce reinjury risk by 84% after ACL reconstruction: the Delaware-Oslo ACL cohort study. Br J Sports Med. 2016;50(13):804-808. 27162233 10.1136/bjsports-2016-096031 PMC4912389

[26] Hambly K Bobic V Wondrasch B Van Assche D Marlovits S. Autologous chondrocyte implantation postoperative care and rehabilitation: science and practice. Am J Sports Med. 2006;34(6):1020-1038. 16436540 10.1177/0363546505281918

[27] Hangody L Kish G Karpati Z Szerb I Udvarhelyi I. Arthroscopic autogenous osteochondral mosaicplasty for the treatment of femoral condylar articular defects: a preliminary report. Knee Surg Sports Traumatol Arthrosc. 1997;5(4):262-267. 9430578 10.1007/s001670050061

[28] Hirschmuller A Baur H Braun S Kreuz PC Südkamp NP Niemeyer P. Rehabilitation after autologous chondrocyte implantation for isolated cartilage defects of the knee. Am J Sports Med. 2011;39(12):2686-2696. 21602564 10.1177/0363546511404204

[29] Ingersoll CD Grindstaff TL Pietrosimone BG Hart JM. Neuromuscular consequences of anterior cruciate ligament injury. Clin Sports Med. 2008;27(3):383-404, vii. 10.1016/j.csm.2008.03.004 18503874

[30] Keays SL Bullock-Saxton JE Newcombe P Keays AC. The relationship between knee strength and functional stability before and after anterior cruciate ligament reconstruction. J Orthop Res. 2003;21(2):231-237. 12568953 10.1016/S0736-0266(02)00160-2

[31] Kon E Di Martino A Filardo G , et al. Second-generation autologous chondrocyte transplantation: MRI findings and clinical correlations at a minimum 5-year follow-up. Eur J Radiol. 2011;79(3):382-388. 20457500 10.1016/j.ejrad.2010.04.002

[32] Kyritsis P Bahr R Landreau P Miladi R Witvrouw E. Likelihood of ACL graft rupture: not meeting six clinical discharge criteria before return to sport is associated with a four times greater risk of rupture. Br J Sports Med. 2016;50(15):946-951. 27215935 10.1136/bjsports-2015-095908

[33] Lysholm J Gillquist J. Evaluation of knee ligament surgery results with special emphasis on use of a scoring scale. Am J Sports Med. 1982;10(3):150-154. 6896798 10.1177/036354658201000306

[34] Marlovits S Singer P Zeller P Mandl I Haller J Trattnig S. Magnetic resonance observation of cartilage repair tissue (MOCART) for the evaluation of autologous chondrocyte transplantation: determination of interobserver variability and correlation to clinical outcome after 2 years. Eur J Radiol. 2006;57(1):16-23. 16203119 10.1016/j.ejrad.2005.08.007

[35] Marlovits S Striessnig G Resinger CT , et al. Definition of pertinent parameters for the evaluation of articular cartilage repair tissue with high-resolution magnetic resonance imaging. Eur J Radiol. 2004;52(3):310-319. 15544911 10.1016/j.ejrad.2004.03.014

[36] Minas T. Autologous chondrocyte implantation for focal chondral defects of the knee. Clin Orthop Relat Res. 2001;391(suppl):S349-S361. 10.1097/00003086-200110001-00032 11603718

[37] Mithoefer K Hambly K Logerstedt D Ricci M Silvers H Della Villa S. Current concepts for rehabilitation and return to sport after knee articular cartilage repair in the athlete. J Orthop Sports Phys Ther. 2012;42(3):254-273. 22383103 10.2519/jospt.2012.3665

[38] Niethammer TR Altmann D Holzgruber M , et al. Patient-reported and magnetic resonance imaging outcomes of third-generation autologous chondrocyte implantation after 10 years. Arthroscopy. 2020;36(7):1928-1938. 32200064 10.1016/j.arthro.2020.03.009

[39] Reinold MM Wilk KE Macrina LC Dugas JR Cain EL. Current concepts in the rehabilitation following articular cartilage repair procedures in the knee. J Orthop Sports Phys Ther. 2006;36(10):774-794. 17063839 10.2519/jospt.2006.2228

[40] Riegger-Krugh CL McCarty EC Robinson MS Wegzyn DA. Autologous chondrocyte implantation: current surgery and rehabilitation. Med Sci Sports Exerc. 2008;40(2):206-214. 18202583 10.1249/mss.0b013e31815cb228

[41] Robertson WB Fick D Wood DJ Linklater JM Zheng MH Ackland TR. MRI and clinical evaluation of collagen-covered autologous chondrocyte implantation (CACI) at two years. Knee. 2007;14(2):117-127. 17257849 10.1016/j.knee.2006.11.009

[42] Roos EM Roos HP Lohmander LS Ekdahl C Beynnon BD. Knee Injury and Osteoarthritis Outcome Score (KOOS)—development of a self-administered outcome measure. J Orthop Sports Phys Ther. 1998;28(2):88-96. 9699158 10.2519/jospt.1998.28.2.88

[43] Schmitt LC Paterno MV Hewett TE. The impact of quadriceps femoris strength asymmetry on functional performance at return to sport following anterior cruciate ligament reconstruction. J Orthop Sports Phys Ther. 2012;42(9):750-759. 22813542 10.2519/jospt.2012.4194 PMC4157226

[44] Steadman JR Rodkey WG Rodrigo JJ. Microfracture: surgical technique and rehabilitation to treat chondral defects. Clin Orthop Relat Res. 2001;391(suppl):S362-S369. 10.1097/00003086-200110001-00033 11603719

[45] Tegner Y Lysholm J. Rating systems in the evaluation of knee ligament injuries. Clin Orthop Relat Res. 1985;198:43-49. 4028566

[46] Trattnig S Pinker K Krestan C Plank C Millington S Marlovits S. Matrix-based autologous chondrocyte implantation for cartilage repair with HyalograftC: Two-year follow-up by magnetic resonance imaging. Eur J Radiol. 2006;57(1):9-15. 16183239 10.1016/j.ejrad.2005.08.006

[47] Wellsandt E Failla MJ Snyder-Mackler L. Limb symmetry indexes can overestimate knee function after anterior cruciate ligament injury. J Orthop Sports Phys Ther. 2017;47(5):334-338. 28355978 10.2519/jospt.2017.7285 PMC5483854

[48] Welsch GH Mamisch TC Zak L , et al. Evaluation of cartilage repair tissue after matrix-associated autologous chondrocyte transplantation using a hyaluronic-based or a collagen-based scaffold with morphological MOCART scoring and biochemical T2 mapping: preliminary results. Am J Sports Med. 2010;38(5):934-942. 20335510 10.1177/0363546509354971

[49] Wondrasch B Risberg MA Zak L Marlovits S Aldrian S. Effect of accelerated weightbearing after matrix-associated autologous chondrocyte implantation on the femoral condyle: a prospective, randomized controlled study presenting MRI-based and clinical outcomes after 5 years. Am J Sports Med. 2015;43(1):146-153. 25378208 10.1177/0363546514554910

[50] Wondrasch B Zak L Welsch GH Marlovits S. Effect of accelerated weightbearing after matrix-associated autologous chondrocyte implantation on the femoral condyle on radiographic and clinical outcome after 2 years: a prospective, randomized controlled pilot study. Am J Sports Med. 2009;37(suppl 1):88S-96S. 10.1177/0363546509351272 19846693

